# Prediction of Protein Mutational Free Energy: Benchmark and Sampling Improvements Increase Classification Accuracy

**DOI:** 10.3389/fbioe.2020.558247

**Published:** 2020-10-08

**Authors:** Brandon Frenz, Steven M. Lewis, Indigo King, Frank DiMaio, Hahnbeom Park, Yifan Song

**Affiliations:** ^1^Cyrus Biotechnology, Seattle, WA, United States; ^2^Department of Biochemistry, University of Washington, Seattle, WA, United States

**Keywords:** mutation, protein, mutation free energy, protein design and engineering, thermodynamics

## Abstract

Software to predict the change in protein stability upon point mutation is a valuable tool for a number of biotechnological and scientific problems. To facilitate the development of such software and provide easy access to the available experimental data, the ProTherm database was created. Biases in the methods and types of information collected has led to disparity in the types of mutations for which experimental data is available. For example, mutations to alanine are hugely overrepresented whereas those involving charged residues, especially from one charged residue to another, are underrepresented. ProTherm subsets created as benchmark sets that do not account for this often underrepresent tense certain mutational types. This issue introduces systematic biases into previously published protocols’ ability to accurately predict the change in folding energy on these classes of mutations. To resolve this issue, we have generated a new benchmark set with these problems corrected. We have then used the benchmark set to test a number of improvements to the point mutation energetics tools in the Rosetta software suite.

## Introduction

The ability to accurately predict the stability of a protein upon mutation is important for numerous problems in protein engineering and medicine including stabilization and activity optimization of biologic drugs. To perform this task a number of strategies and force fields have been developed, including those that perform exclusively on sequence (Casadio et al., 1995; Capriotti et al., 2005; Kumar et al., 2009) as well as those that involve sophisticated physical force fields both knowledge based (Sippl, 1995; Gilis and Rooman, 1996; Potapov et al., 2009), physical models (Pitera and Kollman, 2000; Pokala and Handel, 2005; Benedix et al., 2009), and hybrids (Pitera and Kollman, 2000; Guerois et al., 2002; Kellogg et al., 2011; Jia et al., 2015; Park et al., 2016; Quan et al., 2016).

To facilitate the development of these methodologies and provide easy access to the available experimental information the ProTherm database (Uedaira et al., 2002) was developed. This database collects thermodynamic information on a large number of protein mutations and makes it available in an easy to access format. At the time of this writing it contains 26,045 entries.

Due to its ease of access the ProTherm has served as the starting point for a number of benchmark sets used to validate different stability prediction software packages, including those in the Rosetta software suite. However significant biases exist in the representation of different types and classes of mutations in the ProTherm, as it is derived from the existing literature across many types of proteins and mutations. The most obvious example of this is the large number of entries involving a mutation from a native residue to alanine as making this type of mutation is a common technique used to find residues important for protein function. Therefore a large number of the benchmark sets derived from the ProTherm, which did not account for this bias, have significantly under or overrepresented these classes of mutations. These findings suggest previous reports on the accuracy of stability prediction software does not accurately reflect these tools’ ability to predict stability changes across all classes of mutations.

To address this issue we have generated a novel benchmark subset which accounts for this bias in the ProTherm database (Supplementary Table 1). We then used this benchmark set to validate and improve upon an existing free energy of mutation tool within the Rosetta software suite, “Cartesian ΔΔG,” first described in Park et al. (2016).

## Results

**FIGURE 1:**
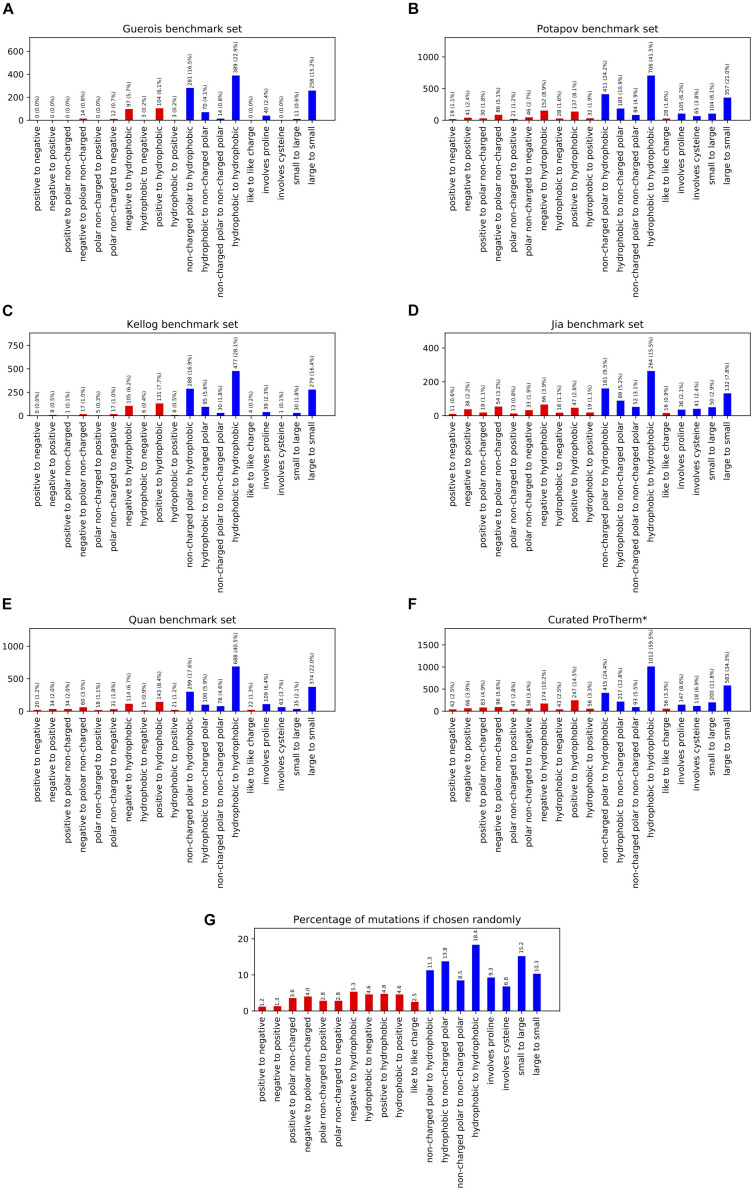
This figure shows the population of different mutation classes used to benchmark a number of methods ability to predict the change in free energy upon mutation. The citations for these benchmark sets are as follows: **(A)** Guerois et al. (2002), **(B)** Potapov et al. (2009), **(C)** Kellogg et al. (2011), **(D)** Jia et al. (2015), **(E)** Quan et al. (2016), curated ProTherm* **(F)** Ó Conchúir et al. (2015). The probability of these classifications occurring given the amino acid composition of the structures in the Curated ProTherm* database are shown in **(G)**. Classes involving charged residues are colored in red. All data sets are significantly biased in their types of mutations present, especially when it comes to mutations to hydrophobics. All data sets contain greater than 27% hydrophobic to hydrophobic mutations vs. the expected 18.4% **(G)**.

In order to benchmark our Rosetta-based stability prediction tools we classified the possible mutations into 17 individual categories as well as reported results on four aggregate categories. We analyzed five previously published benchmark sets to determine their coverage across the different classes of mutations and found them inadequate in a number of categories, especially involving charged residues (Figures 1A–E). For example, the number of data points for mutational types ranged from 0 to 24 for negative to positive, 0 to 50 for positive to negative, 3 to 28 for hydrophobic to negative, and 3 to 44 for hydrophobic to positive entries across the benchmark sets tested. Mutations to and/or from hydrophobic residues dominated the benchmark sets ranging from 75 to 92% of the total entries.

To compare the composition of these benchmark sets to that of the database we examined the curated ProTherm (ProTherm^∗^) provided by Ó Conchúir et al. (2015)^1^ which is a selection of entries containing only mutations which occur on a single chain and provide experimental ΔΔG values (Supplementary Table 2). We find that significant biases still exist here, with several categories having fewer than 50 unique mutations. These include: positive to negative, 42; hydrophobic to negative, 43; and non-charged polar to positive, 47. Mutations involving hydrophobic residues are still overrepresented, with 62.2% of all mutations in the database being mutations to hydrophobic residues, compared to the expected 39.8% if mutations from the starting structures were chosen randomly (Figures 1F–G).

We also analyzed the benchmark sets with respect to the number of buried vs. exposed residues in the data sets. No large biases were observed. All benchmark sets were within 6% of what would be expected if mutations were random (data not shown).

To sample more broadly across all types of mutations and remove sources of bias in our algorithm development we created a new benchmark set of single mutations that are more balanced across mutational types and avoid other biases. To generate this set we performed the following operations:

(1) Removed any entries from the curated ProTherm^∗^ that occur on the interface of a protein complex or interact with ligands—the energetics of these mutations would include intra-protein and inter-molecular interactions that would alter the desired intra-protein energetics of a free energy calculation.
(2) We removed entries of identical mutation on similar-sequence (>60%) backbones. For mutations occurring at the same position in similar sequences, if the mutation is identical (e.g., L → I) and the sequence identity >60%, then that mutation is included only once in the database; if the mutation is not identical (L → I in one protein and L → Q in another) then the mutation is included.
(3) We populated each mutation category, excluding small to large, large to small, buried, and surface, with 50 entries except for the cases where insufficient experimental data points exist. Statistics on the excluded categories were derived from data points that were already present in the other categories.
(4) When multiple experimental values (including identical mutations as identified in point 2 above) were available we chose the ΔΔG value taken at the pH closest to 7.

**FIGURE 2:**
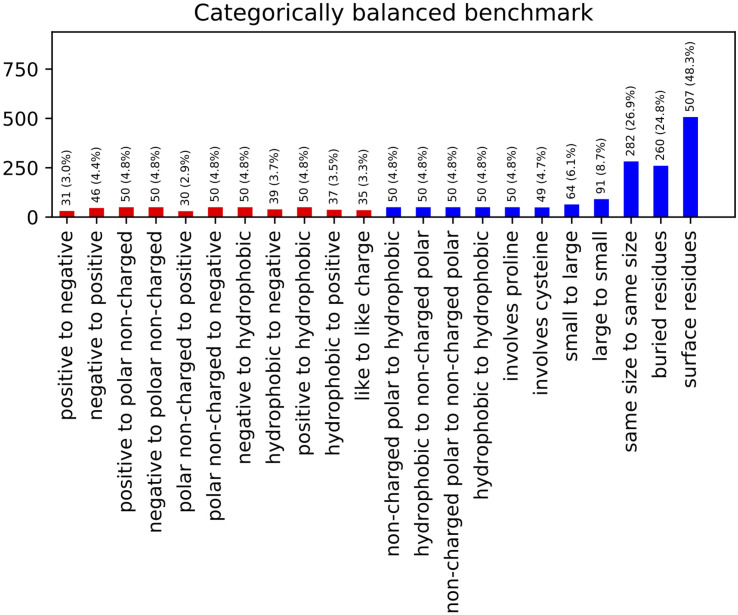
Categorically Balanced Benchmark mutational category statistics. This figure shows the metrics of our new benchmark set selected to provide a more balanced representation of different mutation classifications. Classes involving charged residues are shown in red.

The resulting benchmark set contains 767 entries across a range of different types and classes of mutations (Figure 2). This constitutes a reduction from the 2,971 total entries in the curated ProTherm^∗^, with mutations to hydrophobics being the most frequently being eliminated. This reduction does eliminate potentially useful data, and introduces a slight bias toward solvent exposed residues: 66% of the mutations being on residues with greater than 20% solvent exposed surface area compared to 54% if chosen randomly. This change is useful to reduce bias toward favoring hydrophobic mutations and has been controlled for by checking our algorithm’s performance when residues are classified by burial.

We tested Protocol 3 described in Kellogg et al. (2011) on this benchmark set (Table 1). To assess method quality, we analyzed prediction power by a number of different methods including Pearson’s R, Predictive Index (Pearlman and Charifson, 2001), and Matthews Correlation Coefficient (MCC) (Matthews, 1975). We also analyzed classification errors instead of correlation. A mutation is classified as stabilizing if the change in free energy is ≤-1 kcal/mol, it is classified as destabilizing if the change is ≥1 kcal/mol, and neutral if it falls between these values. Each mutation is assigned a value of 0 for destabilizing, 1 for neutral, and 2 for stabilizing. We then scored each entry by taking the absolute value of the difference between the value for the experiment and the prediction. A value of 0 indicates the prediction was correct, 1 indicates the prediction was moderately incorrect, i.e., the mutation is destabilizing and the prediction was neutral, and 2 indicates the prediction was egregiously wrong.

**TABLE 1:** Correlations and Predictive Index for Protocol 3 and our improved Cartesian ΔΔG across different mutation categories.

	Protocol 3				Cartesian ΔΔG			
Mutation Type	Pearson’s R	Pearson’s R Filtered	Predictive Index	MCC	Pearson’s R	Pearson’s R Filtered	Predictive Index	MCC
Small to large	0.54 ± 0.000	0.68 ± 0.000	0.57 ± 0.001	0.36 ± 0	0.48 ± 0.0041	0.66 ± 0.006	0.55 ± 0.0096	0.55 ± 0.0221
Large to small	0.57 ± 0.000	0.76 ± 0.000	0.59 ± 0.000	0.37 ± 0	0.62 ± 0.0199	0.8 ± 0.0015	0.71 ± 0.0239	0.46 ± 0.0176
Positive to negative	0.40 ± 0.000	0.79 ± 0.000	0.28 ± 0.000	0.00 ± 0	0.65 ± 0.0024	0.88 ± 0.0072	0.74 ± 0.0054	0.5 ± 0.0891
Negative to positive	0.34 ± 0.000	0.61 ± 0.000	0.26 ± 0.000	0.19 ± 0	0.36 ± 0.0128	0.57 ± 0.0176	0.48 ± 0.0312	0.53 ± 0.0302
Negative to hydrophobic	0.27 ± 0.000	0.55 ± 0.000	0.27 ± 0.000	0.15 ± 0	0.58 ± 0.0064	0.71 ± 0.0124	0.64 ± 0.0068	0.43 ± 0.0228
Hydrophobic to negative	0.83 ± 0.000	0.87 ± 0.000	0.84 ± 0.000	0.27 ± 0	0.73 ± 0.0554	0.81 ± 0.0477	0.8 ± 0.0867	0.5 ± 0.0551
Positive to hydrophobic	0.06 ± 0.000	0.23 ± 0.000	0.01 ± 0.000	0.35 ± 0	0.46 ± 0.0266	0.62 ± 0.0326	0.51 ± 0.0283	0.66 ± 0.0016
Hydrophobic to positive	0.57 ± 0.000	0.73 ± 0.000	0.63 ± 0.002	0.37 ± 0	0.51 ± 0.0112	0.7 ± 0.0031	0.67 ± 0.0074	0.44 ± 0.0858
Non-charged polar to positive	0.40 ± 0.000	0.67 ± 0.000	0.39 ± 0.004	0.43 ± 0	0.28 ± 0.0075	0.78 ± 0.0148	0.4 ± 0.0196	0.28 ± 0.0
Positive to non-charged polar	0.32 ± 0.000	0.67 ± 0.000	0.52 ± 0.000	0.26 ± 0	0.43 ± 0.0042	0.8 ± 0.0178	0.72 ± 0.0084	0.55 ± 0.0602
Non-charged polar to negative	0.64 ± 0.000	0.73 ± 0.000	0.69 ± 0.000	0.00 ± 0	0.62 ± 0.0196	0.83 ± 0.0042	0.66 ± 0.0153	0.67 ± 0.0
Negative to non-charged polar	0.13 ± 0.000	0.44 ± 0.000	-0.07 ± 0.000	0.22 ± 0	0.37 ± 0.0076	0.69 ± 0.0138	0.44 ± 0.0135	0.53 ± 0.028
Non-charged polar to hydrophobic	0.70 ± 0.000	0.70 ± 0.000	0.64 ± 0.001	0.38 ± 0	0.74 ± 0.0014	0.78 ± 0.0012	0.66 ± 0.0027	0.38 ± 0.0
Hydrophobic to non-charged polar	0.41 ± 0.000	0.66 ± 0.000	0.39 ± 0.000	0.47 ± 0	0.57 ± 0.0105	0.75 ± 0.022	0.58 ± 0.0027	0.11 ± 0.022
Non-charged polar to non-charged polar	0.76 ± 0.000	0.76 ± 0.000	0.66 ± 0.002	0.15 ± 0	0.52 ± 0.0049	0.84 ± 0.0038	0.79 ± 0.0021	0.49 ± 0.0313
Hydrophobic to hydrophobic	0.67 ± 0.000	0.74 ± 0.000	0.72 ± 0.000	0.57 ± 0	0.61 ± 0.0051	0.75 ± 0.0068	0.68 ± 0.0111	0.28 ± 0.0282
charge to charge	0.31 ± 0.000	0.73 ± 0.000	0.36 ± 0.000	0.26 ± 0	0.32 ± 0.0067	0.7 ± 0.0143	0.35 ± 0.0055	0.44 ± 0.0397
Involves cysteine	0.25 ± 0.000	0.63 ± 0.000	0.27 ± 0.000	0.26 ± 0	0.07 ± 0.0428	0.49 ± 0.0946	0.16 ± 0.0498	0.08 ± 0.0708
Involves proline	0.02 ± 0.000	0.54 ± 0.000	0.33 ± 0.000	0.30 ± 0	0.51 ± 0.0277	0.76 ± 0.0264	0.54 ± 0.0271	0.51 ± 0.1401
Same size	0.36 ± 0.000	0.38 ± 0.000	0.37 ± 0.000	0.22 ± 0	0.45 ± 0.0035	0.45 ± 0.0035	0.51 ± 0.0091	0.31 ± 0.0251
Buried	0.20 ± 0.000	0.54 ± 0.000	0.55 ± 0.000	0.35 ± 0	0.43 ± 0.0022	0.43 ± 0.0022	0.54 ± 0.0104	0.26 ± 0.0056
Surface	0.31 ± 0.000	0.34 ± 0.000	0.35 ± 0.000	0.23 ± 0	0.47 ± 0.006	0.5 ± 0.0064	0.6 ± 0.0076	0.42 ± 0.0165
Everything	0.25 ± 0.000	0.47 ± 0.000	0.48 ± 0.000	0.28 ± 0	0.49 ± 0.0025	0.49 ± 0.0025	0.61 ± 0.0062	0.41 ± 0.0127

*This table contains the Pearson’s R correlations for each class of mutations in our benchmark set for both Protocol 3 and Cartesian ΔΔG. Each is repeated three times using the same inputs and the average and standard deviation are shown. Given the sensitivity to outliers of Pearson’s R we also report it as Pearson’s R filtered after removing up to five outliers from each set. An outlier is defined as any single entry which, when removed, changes the correlation coefficient by 0.025 or greater. We also report the Predictive Index and the Matthews Correlation Coefficient which are less sensitive to the absolute free energy of a prediction but rather whether it can be correctly classified. Cartesian ΔΔG significantly outperforms Protocol 3 both in the unfiltered Pearson’s R, Predictive Index, and Matthews Correlation Coefficient. In each analysis metric the higher value indicates greater accuracy.*

To address some metrics on which Protocol 3 performs poorly, we were interested in using a more modern Rosetta ΔΔG protocol, Cartesian ΔΔG, first briefly described in Park et al. (2016). We refactored the Cartesian ΔΔG code to utilize the Mover framework described in Leaver-Fay et al. (2011), keeping the underlying science the same (other than changes highlighted here) while eliminating bugs and improving efficiency and modifiability (Supplementary Table 3).

**FIGURE 3:**
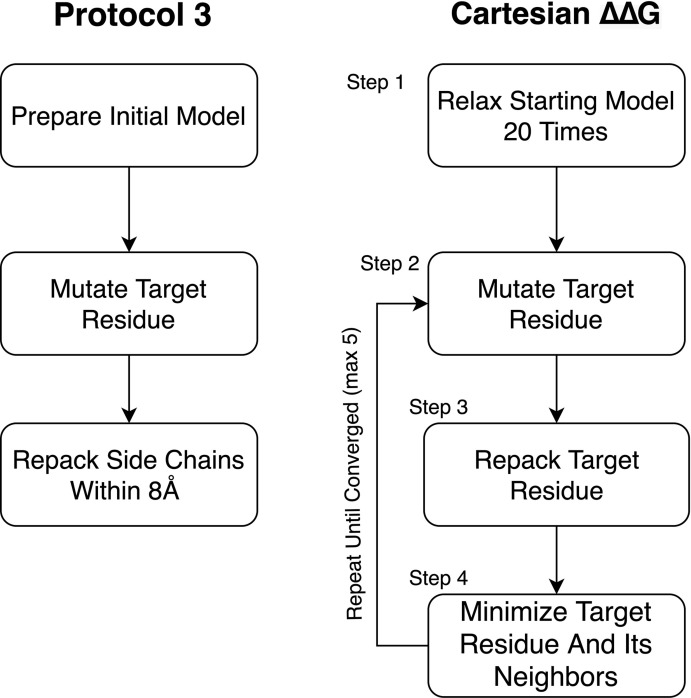
Diagram of Protocol 3 and Cartesian ΔΔG. This figure diagrams the steps involved in the older Protocol 3 as well as the Cartesian ΔΔG protocol. Novel changes described in this paper include the removal of constraints during step 1 of the Cartesian ΔΔG protocol, the addition of Step 2.1 for mutations involving proline as well as the choice to repeat testing until the protocol converges on a lower energy score instead of a fixed number (3) of times.

We tested changes to the preparation phase of the protocol relative to what was used in Park et al. (2016; Figure 3). To improve the preparation step (step 1), we tested Cartesian Relax (as opposed to traditional torsion space Relax, used in Kellogg’s Protocol 3) (Kellogg et al., 2011) both with and without all atom constraints and found that Pearson’s R correlations were worse when models were prepared without constraints but the Predictive Index and MCC improved. The variability between runs also dropped when models were prepared without constraints. The biggest impact was on the classification of mutations, however, with the number of egregiously wrong predictions falling from 34.00 ± 2.0 to 24.67 ± 0.6 (Supplementary Table 2). This likely has to do with the use of Cartesian minimization during step 4, and the importance of preparing a structure with similar sampling methods to those used during mutational energy evaluation. We consider the MCC and Predictive Index improvements more valuable and thus recommend model preparation without all atom constraints.

We also examined a potential runtime improvement for Cartesian ΔΔG. In Park et al. (2016), the final energy for a mutation is the average of three replicates. We examined a multi-run convergence criterion, described in further detail below, and settled on the convergence criterion method due to its equivalent accuracy with reduced run time.

Finally, we tested adding increased backbone sampling around residues that are being mutated to or from proline, which had no impact on the Pearson’s R, Predictive Index, and MCC, but reduced the number of egregious errors slightly from 25.33 ± 0.6 to 24.67 ± 0.6 (Supplementary Table 3).

This updated Cartesian ΔΔG algorithm has improved performance overall when compared to Protocol 3, especially in the ability to accurately classify mutations including the large reduction of egregious errors in classification (Tables 1, 2). For example, the number of mutations predicted as stabilizing when they are destabilizing or vice versa fell from an average of 53 with Protocol 3 to an average of 24.6 across three replicates. “Off by 1” errors are also lower (317.3 vs. 289.3) (Supplementary Table 2). This trend is much stronger than the improvement in correlations, and more importantly reflects the practical value of correctly classifying mutational categories. For example in protein engineering, a protein designer’s practical interest is whether any given mutation is stabilizing at all, more than which of two mutations is more stabilizing.

**TABLE 2:** The ability of Protocol 3 and Cartesian ΔΔG to correctly classify mutations.

	Protocol 3			Cartesian ΔΔG			
Mutation class	Same class (%)	Off by one (%)	Off by two (%)	Same class (%)	Off by one (%)	Off by two (%)	Total entries
Small to large	55.7	42.7	1.6	67.7	29.2	3.1	64
Large to small	60.4	34.1	5.5	53.5	42.9	3.7	91
Positive to negative	32.3	61.3	6.5	67.7	32.3	0.0	31
Negative to positive	26.1	56.5	17.4	55.1	35.5	9.4	46
Negative to hydrophobic	32.0	50.0	18.0	56.0	38.0	6.0	50
Hydrophobic to negative	53.8	43.6	2.6	70.9	29.1	0.0	39
Positive to hydrophobic	50.0	42.0	8.0	47.3	50.0	2.7	50
Hydrophobic to positive	73.0	18.9	8.1	79.3	18.0	2.7	37
Non-charged polar to positive	50.0	50.0	0.0	61.1	35.6	3.3	30
Positive to non-charged polar	58.0	38.0	4.0	61.3	36.7	2.0	50
Non-charged polar to negative	48.0	48.0	4.0	58.0	40.0	2.0	50
Negative to non-charged polar	44.0	36.0	20.0	50.7	47.3	2.0	50
Non-charged polar to hydrophobic	48.0	48.0	4.0	48.7	49.3	2.0	50
Hydrophobic to non-charged polar	56.0	42.0	2.0	66.0	28.7	5.3	50
Non-charged polar to non-charged polar	57.3	38.7	4.0	67.3	30.7	2.0	50
Hydrophobic to hydrophobic	76.0	22.0	2.0	72.0	26.0	2.0	50
Charge to charge	65.7	34.3	0.0	51.4	48.6	0.0	35
Involves cysteine	55.1	40.8	4.1	49.3	44.0	6.7	49
Involves proline	54.0	38.0	8.0	52.0	44.0	4.0	50
Buried	65.4	26.5	8.1	65.0	29.4	5.6	260
Surface	44.7	49.0	6.3	56.1	41.9	2.0	507
Everything	51.7	41.4	6.9	59.1	37.7	3.2	767

*This table shows the ability of Protocol 3 and Cartesian ΔΔG to correctly classify a mutation. Mutations are assigned a value of 0 for destabilizing, 1 for neutral, and 2 for stabilizing. The absolute value of the difference between the predicted class and the experimental class represents no error, mild error (off by one class), or egregious error (off by two classes). Performance across the benchmark is reported here as a percentage of mutations in each class. Cartesian ΔΔG correctly classifies more entries (59.1 vs. 51.7%), and produces fewer catastrophic off by two errors (3.2 vs. 6.9%).*

The overall level of accurate classification predictions increases from 51.7 to 59.1% from the Protocol 3 to Cartesian ΔΔG Rosetta methods. We also note that over all charged residues the category predictions accuracy was 47.9% for protocol 3 and increases to over 60.5% with Cartesian ΔΔG. The Cartesian ΔΔG algorithm is more broadly useful across any type of protein mutation, while Protocol 3 had uneven applicability.

## Discussion

Here we describe a number of issues in previous benchmark sets used to assess the quality of protein stability prediction software. In particular we have found a lack of adequate experimental data being included for mutations involving charged residues.

Using these updated benchmarks we show that protein stability prediction tools in Rosetta vary widely across different types of mutation classes. In addition, given that this problem is pervasive throughout the field, it is likely that the reported accuracy of many methods for stability prediction may not reflect the diversity of possible mutation types. We encourage other developers to analyze the performance of their tools across different types of mutations using our benchmark set or one which has appropriately accounted for the biases that exist within the databases (Supplementary Table 1). The reduced size of this data set may also be useful for rapid training or situations with computational limitations.

Last, we have refactored the Cartesian ΔΔG protocol code to improve consistency and modifiability, and have also made minor modifications to the structure preparation and analysis step as well as to how mutations involving proline are sampled. By analyzing these algorithms with the new benchmark set, and focusing on previously underrepresented categories of mutations (e.g., uncharged to charged), we are able to demonstrate the Cartesian ΔΔG algorithm has improved correlation to experimental values and improved ability to correctly classify (stabilizing/destabilizing/neutral) a mutation relative to the older Protocol 3 methodology. These results show the importance of diverse datasets in algorithm benchmarking, and the need to look beyond the surface when analyzing the results of these algorithms.

## Methods

### Benchmark Set Pruning

To create our benchmark set, we began by making a copy of the curated ProTherm^∗^ database (Ó Conchúir et al., 2015) and began removing entries that were unsuitable. Because we wished to develop a point mutation algorithm without the complexities of multiple mutation interactions, we excluded any entry which did not represent a single mutation. Because the algorithm is intended to represent ΔΔG of monomer folding and not binding interactions, we also removed entries on the interface of a protein-protein complex, or interacting with a non-water ligand. Interactions were defined as any atom in the mutated residue within 5 Å of an atom not on the same chain. To increase experimental diversity, we wished to remove duplicate mutations. To identify duplicates, we performed an all to all sequence alignment to find parent backbones with ≥60% sequence identity. Within these clusters of sequences, any entries in which the same native residue is mutated to the same target were treated as identical. When multiple experimental ΔΔG values were available for an identical mutation we chose the value taken at closer to neutral pH.

### Benchmark Category Population

We identified 21 categories of mutation type by combinations from nine residue type classifications (Table 3). We then populated each narrowly defined category (e.g., polar non-charged to negative) with up to 50 entries. Some categories (large to small, small to large, buried, and surface) are supersets of the more narrowly defined categories and were sufficiently populated by the experiments selected from the other groups.

**TABLE 3:** Residue category assignments and category combinations.

Type and 1 letter codes	Combination categories	
Small GAVSTC	Positive to negative	Non-charged polar to hydrophobic
Large FYWKRHQE	Negative to positive	Hydrophobic to non-charged polar
Negative DE	Positive to non-charged polar	Non- charged polar to non-charged polar
Positive RK	Negative to non-charged polar	Hydrophobic to hydrophobic
Polar YTSHKREDQN	Non-charged polar to positive	Like to like charge
Non-charged polar YTSNQH	Non-charged polar to negative	Involves proline
Hydrophobic FILVAGMW	Negative to hydrophobic	Involves cysteine
Cysteine C	Hydrophobic to negative	Small to large
Proline P	Positive to hydrophobic	Large to small
	Hydrophobic to positive	

*On the left, we list the nine residue groupings considered in this benchmark and annotate which residues go in each class. At center and right, we list the 19 mutational types considered by combining these classes.*

A few categories involving charged residues (positive to negative, negative to positive, non-charged polar to positive, hydrophobic to negative, hydrophobic to positive, and like to like charge) did not have enough data to hit 50 entries so every available unique experiment was added.

### ΔΔG Prediction

To prepare models for ΔΔG calculations, structures were stripped to only the chain in which the mutation occurs. Rosetta local refinement, consisting of alternating cycles of side chain packing and all atom minimization (“Relax”), was then performed 20 times on the chain of interest and the model with the lowest Rosetta energy was selected as input. As noted in the Results, this was done without all atom constraints and in Cartesian space, not torsional space.

ΔΔG predictions were then performed using Protocol 3 described in Kellogg et al. (2011), the version of the Cartesian ΔΔG application described originally in Park et al. (2016), or the refactored and improved version of Cartesian ΔΔG elaborated upon here.

To provide context for our modifications, a brief description of the Cartesian ΔΔG protocol as presented in Park et al. (2016) is warranted. Cartesian ΔΔG calculates the change in folding energy upon mutation by taking the prepared starting structures, then mutating the target residue. This residue and its neighbors within 6 Å are then repacked. After repacking the mutated residue, the side chain atoms of residues within 6 Å of the target residue and the side chain and backbone atoms of sequence-adjacent residues are minimized in Cartesian space. The same optimization, without the change in sequence, is done on the starting structure to determine the baseline energy. The process is performed three times for both the mutant and the wild type sequence and the ΔΔG is calculated from the average of each. There is no particularly different handling of mutations involving proline.

Our modifications to the Cartesian ΔΔG tool include a change to the analysis and a change to proline handling (Figure 2). In the analysis step, we changed the number of mutant models generated using the following convergence criterion: the lowest energy 2 structures must converge to within 1 Rosetta Energy Unit, or take the best of 5 models, whichever comes first. In either case the lowest, not the average, energy is used. In order to address changes in the backbone resulting from mutations to and from proline we added additional fragment based sampling around mutations involving proline. By default 30 fragments of 5 residues in length, centered on the mutation, are sampled and the best scoring structure is carried forward for analysis. This uses the Cartesian Sampler system described in Wang et al. (2016). Command line flags and XML files can be found in Supplementary Material.

## Data Availability Statement

All datasets generated for this study are included in the article/Supplementary Material. The protocol and source code are freely available for academic use in the Rosetta software suite found at: https://www.rosettacommons.org/.

## Author Contributions

BF carried out the primary work and prepared the manuscript. SL analyzed data and edited the manuscript. IK analyzed data. FD and HP helped develop the initial code and provided guidance. YS supervised the work and provided assistance. All authors contributed to the article and approved the submitted version.

## Conflict of Interest

Cyrus Biotechnology, Inc. funded and designed this study, was responsible for collection, analysis, and interpretation of the data as well as the writing of this article and decision to submit it for publication. They also provided software and infrastructure and employed BF, SL, IK, and YS. The product Cyrus Bench was currently marketed and uses software and methods developed in this study. The remaining authors declare that the research was conducted in the absence of any commercial or financial relationships that could be construed as a potential conflict of interest.

## Footnotes

1 https://guybrush.ucsf.edu/benchmarks/benchmarks/DDG

## References

[B1] Benedix A. Becker C. M. de Groot B. L. Caflisch A. Böckmann R. A. (2009). Predicting free energy changes using structural ensembles. *Nat. Methods* 6 3–4. 10.1038/nmeth0109-3 19116609

[B2] Capriotti E. Fariselli P. Casadio R. (2005). I-Mutant2.0: predicting stability changes upon mutation from the protein sequence or structure. *Nucleic Acids Res.* 33 W306–W310. 15980478 10.1093/nar/gki375 PMC1160136

[B3] Casadio R. Compiani M. Fariselli P. Vivarelli F. (1995). Predicting free energy contributions to the conformational stability of folded proteins from the residue sequence with radial basis function networks. *Proc. Int. Conf. Intell. Syst. Mol. Biol.* 3 81–88. 7584470

[B4] Frenz B. Lewis S. King I. Park H. DiMaio F. Song Y. (2020). Prediction of protein mutational free energy: benchmark and sampling improvements increase classification accuracy. *bioRxiv* [Preprint] 10.1101/2020.03.18.989657 PMC7579412 33134287

[B5] Gilis D. Rooman M. (1996). Stability changes upon mutation of solvent- accessible residues in proteins evaluated by database-derived potentials. *J. Mol. Biol.* 257 1112–1126. 10.1006/jmbi.1996.0226 8632471

[B6] Guerois R. Nielsen J. E. Serrano L. (2002). Predicting changes in the stability of proteins and protein complexes: a study of more than 1000 mutations. *J. Mol. Biol.* 320 369–387. 10.1016/s0022-2836(02)00442-4 12079393

[B7] Jia L. Yarlagadda R. Reed C. C. (2015). Structure based thermostability prediction models for protein single point mutations with machine learning tools. *PLoS One* 10:e0138022. 10.1371/journal.pone.0138022 26361227 PMC4567301

[B8] Kellogg E. H. Leaver-Fay A. Baker D. (2011). Role of conformational sampling in computing mutation-induced changes in protein structure and stability. *Proteins* 79 830–838. 10.1002/prot.22921 21287615 PMC3760476

[B9] Kumar P. Henikoff S. Ng P. C. (2009). Predicting the effects of coding non-synonymous variants on protein function using the SIFT algorithm. *Nat. Protoc.* 4 1073–1081. 10.1038/nprot.2009.86 19561590

[B10] Leaver-Fay A. Tyka M. Lewis S. M. Lange O. F. Thompson J. Jacak R. (2011). Rosetta3: an object-oriented software suite for the simulation and design of macromolecules. *Methods Enzymol.* 487 545–574. 21187238 10.1016/B978-0-12-381270-4.00019-6 PMC4083816

[B11] Matthews B. W. (1975). Comparison of the predicted and observed secondary structure of T4 phage lysozyme. *Biochim. Biophys. Acta* 405 442–451. 10.1016/0005-2795(75)90109-9 1180967

[B12] Ó Conchúir S. Barlow K. A. Pache R. A. Ollikainen N. Kundert K. O’Meara M. J. (2015). A web resource for standardized benchmark datasets, metrics, and rosetta protocols for macromolecular modeling and design. *PLoS One* 10:e0130433. 10.1371/journal.pone.0130433 26335248 PMC4559433

[B13] Park H. Bradley P. Greisen P. Jr. Liu Y. Mulligan V. K. Kim D. E. (2016). Simultaneous optimization of biomolecular energy functions on features from small molecules and macromolecules. *J. Chem. Theory Comput.* 12 6201–6212. 10.1021/acs.jctc.6b00819 27766851 PMC5515585

[B14] Pearlman D. A. Charifson P. S. (2001). Are free energy calculations useful in practice? A comparison with rapid scoring functions for the p38 MAP kinase protein system†. *J. Med. Chem.* 44 3417–3423. 10.1021/jm0100279 11585447

[B15] Pitera J. W. Kollman P. A. (2000). Exhaustive mutagenesis in silico: multicoordinate free energy calculations on proteins and peptides. *Proteins* 41 385–397. 10.1002/1097-0134(20001115)41:3<385::aid-prot100>3.0.co;2-r 11025549

[B16] Pokala N. Handel T. M. (2005). Energy functions for protein design: adjustment with protein-protein complex affinities, models for the unfolded state, and negative design of solubility and specificity. *J. Mol. Biol.* 347 203–227. 10.1016/j.jmb.2004.12.019 15733929

[B17] Potapov V. Cohen M. Schreiber G. (2009). Assessing computational methods for predicting protein stability upon mutation: good on average but not in the details. *Protein Eng. Des. Sel.* 22 553–560. 10.1093/protein/gzp030 19561092

[B18] Quan L. Lv Q. Zhang Y. (2016). STRUM: structure-based prediction of protein stability changes upon single-point mutation. *Bioinformatics* 32 2936–2946. 10.1093/bioinformatics/btw361 27318206 PMC5039926

[B19] Sippl M. J. (1995). Knowledge-based potentials for proteins. *Curr. Opin. Struct. Biol.* 5 229–235. 10.1016/0959-440x(95)80081-6 7648326

[B20] Uedaira H. Gromiha M. M. Kitajima K. Sarai A. (2002). ProTherm: thermodynamic database for proteins and mutants. *Seibutsu Butsuri Kagaku* 42 276–278. 10.2142/biophys.42.276

[B21] Wang R. Y.-R. Song Y. Barad B. A. Cheng Y. Fraser J. S. DiMaio F. (2016). Automated structure refinement of macromolecular assemblies from cryo-EM maps using Rosetta. *eLife* 5:e17219. 10.7554/eLife.17219 27669148 PMC5115868

